# Gray matter volume moderates the effect of acute physical activity on cognitive load and relates to reading comprehension in adolescents: the Cogni-Action Project

**DOI:** 10.3389/fpsyg.2026.1930058

**Published:** 2026-10-06

**Authors:** Carlos Cristi-Montero, Ricardo Martínez-Flores, Hans Supèr, Javier Sanchez-Martinez, Patricio Solis-Urra, Benjamin Tari, Romualdo Ibáñez, Fabian Herold, Fred Paas, Myrto Mavilidi, Liye Zou

**Affiliations:** 1 IRyS Group, Physical Education School, Pontificia Universidad Católica de Valparaíso, Valparaíso, Chile; 2 Center for Interdisciplinary Research in Biomedicine, Biotechnology and Well-Being (CID3B), Pontificia Universidad Católica de Valparaíso, Valparaíso, Chile; 3 Institute of Neurosciences, University of Barcelona (UBNeuro), Barcelona, Spain; 4 Vision and Control of Action Group, Department of Cognition, Development and Educational Psychology, University of Barcelona, Barcelona, Spain; 5 Catalan Institution for Research and Advanced Studies (ICREA), Barcelona, Spain; 6 Department of Physical Education and Sports, Faculty of Sport Sciences, Sport and Health University Research Institute (iMUDS), University of Granada, Granada, Spain; 7 AdventHealth Research Institute, Neuroscience Institute, Orlando, FL, United States; 8 Faculty of Education and Social Sciences, Universidad Andres Bello, Viña del Mar, Chile; 9 Institute of Sport, Exercise & Health Division of Surgery & Interventional Science, University College London, London, United Kingdom; 10 Instituto de Literatura y Ciencias del Lenguaje, Pontificia Universidad Católica de Valparaíso, Millennium Nucleus for the Science of Learning (MiNSoL), Valparaíso, Chile; 11 Department of Physiology, Faculty of Medicine, HMU Health and Medical University Erfurt, Erfurt, Germany; 12 Department of Psychology, Education and Child Studies, Erasmus University Rotterdam, Rotterdam, Netherlands; 13 School of Education, UNSW, Sydney, NSW, Australia; 14 Faculty of Education, & Physical Activity, Sport & Exercise (PASER) Research Theme, Faculty of Health, Southern Cross University, Sydney, NSW, Australia; 15 Body-Brain-Mind Laboratory, School of Psychology, Shenzhen University, Shenzhen, China

**Keywords:** adolescent, brain volume, crossover trial, exercise, pupillometry

## Abstract

**Background:**

Acute physical activity reduces cognitive load (CL) and improves reading comprehension; however, the role of gray matter volume in these effects is unknown. This study examined how gray matter volume moderates the effects of physical activity on cognitive load, and explored its association with reading comprehension.

**Methods:**

A total of 13 male adolescents (aged 12–13 years), a neuroimaging subsample of a larger cohort, completed a randomized crossover trial that involved three energy-matched conditions: sedentary condition (SC), moderate-intensity continuous training (MICT), and cooperative high-intensity interval training (C-HIIT). We measured gray matter volume in the hippocampus, pars opercularis, and brainstem using MRI. The primary outcome cognitive load (pupil dilation) was analyzed using within-subject permutation with max-*T* correction and bootstrapping. Reading comprehension served as a secondary, exploratory outcome.

**Results:**

Physical activity was a significant predictor of cognitive load (*p* < 0.001). Comprehension improved for the C-HIIT condition compared to SC; gray matter volume alone did not predict these results. Volume significantly moderated cognitive load in a region-specific pattern: hippocampal volume amplified the cognitive load increases associated with C-HIIT [*β* = 0.26, 95% CI (0.21, 0.30)], while pars opercularis and brainstem volumes reduced the cognitive load (*β* = −0.30 to −0.32, *p* < 0.001); C-HIIT resulted in the lowest absolute cognitive load. Reading comprehension was higher under C-HIIT compared to SC (*g* = 0.87) and MICT (*g* = 0.50), with gains exploratorily associated with the pars opercularis volume (rho = 0.53–0.59).

**Conclusion:**

Gray matter volume influenced cognitive load through its interaction with physical activity modality, with C-HIIT producing the lowest cognitive load. Comprehension gains under C-HIIT were exploratorily associated with pars opercularis volume, suggesting that specific neuroanatomical characteristics may complement activity modality for cognitive benefits in school settings.

## 1 Introduction

Appropriate cognitive development, including reading comprehension, is critical to acquiring essential skills for later-life success and mental health (John et al., 2022; Eyre et al., 2024). Reading comprehension is a multifaceted process involving not only basic decoding but also the integration of information into coherent mental representations. According to the construction–integration model, it unfolds as a structured process of increasing cognitive complexity (Van Den Broek and Kendeou, 2024; Follmer, 2018; Kintsch, 1988). Accumulating evidence indicates a decline in reading performance among children and adolescents globally (OECD, 2023). The latest “Survey of Adult Skills” by the Organisation for Economic Co-operation and Development (OECD) highlights a persistent decrease in foundational reading abilities (OECD, 2024) and Programme for International Student Assessment (PISA) results show that nearly 20% of 15-year-old students fail to reach baseline reading proficiency (OECD, 2023). Furthermore, poor reading skills during early life are also associated with a higher risk of cognitive decline and dementia (John et al., 2022). Supporting optimal cognitive development early in life is therefore important to foster societal development in line with the United Nations’ Sustainable Development Goals (Eyre et al., 2024).

Individual differences in brain structure, particularly gray matter volume in the bilateral hippocampus, left pars opercularis, and brainstem, serve as robust predictors of reading comprehension, especially during adolescence, when these regions are maturing and susceptible to environmental influences such as physical activity (Keller et al., 2024; Skoe et al., 2017). The brainstem is closely connected to classical biomarkers of text processing, such as pupil dilation, a reliable proxy for cognitive load (CL): Larger dilation indicates higher CL, whereas constriction reflects lower CL (Zou et al., 2023; Joshi and Gold, 2020). Given the embryological link between the eye and brain, pupil size reflects neural activity during cognitive tasks across multiple brain regions, reinforcing the relevance of pupillometry in developmental cognitive neuroscience research due to its non-invasive nature and suitability for children and adolescents (Zou et al., 2023; Joshi and Gold, 2020; Moore et al., 2015; Eckstein et al., 2017).

The brainstem houses several key structures that regulate eye movements and pupil size, including the locus coeruleus, the brain’s primary source of norepinephrine and is crucial for cognitive arousal (Khroud et al., 2024). Through its widespread neural projections, the locus coeruleus modulates cognitive functions related to reading, such as visual attention (Aston-Jones et al., 1999). Functionally, it is connected to the Edinger–Westphal nucleus, which mediates pupillary constriction by relaying signals to the iris sphincter muscle via the ciliary ganglion (Heiland Hogan et al., 2025). Moreover, the locus coeruleus receives excitatory input from the ventral hippocampus, which communicates with the medial prefrontal cortex to regulate attention and information processing (Tsetsenis et al., 2022; van den Brink et al., 2019). Thus, pupil dilation is a relatively easy-to-capture readout of the activation of specific brainstem regions and other brain structures such as the hippocampus and the inferior frontal gyrus, including the pars opercularis, indicating that dilation or constriction relates to increases or decreases in CL (Heiland Hogan et al., 2025; Tsetsenis et al., 2022; Alnæs et al., 2014).

High-intensity physical activity activates the locus coeruleus via its noradrenergic pathway, potentially enhancing attentional control (Vazey et al., 2018; Plini et al., 2024). A single bout of physical activity can enhance brain function (Zou et al., 2023; Yu et al., 2021; Pontifex et al., 2019), although effects depend on mode and intensity, underscoring the importance of a dose–response relationship (Herold et al., 2019; Mavilidi et al., 2025). Such activity has been associated with improved connectivity in brain regions involved in CL and reading comprehension, including the hippocampus and inferior frontal gyrus (Festa et al., 2023).

A recent study found that the effects of physical activity on reading comprehension and CL varied depending on mode and intensity (Martínez-Flores et al., 2025). Specifically, high-intensity interval training was associated with modulated pupillary responses, indicating lower CL during reading and better comprehension performance targeting both surface code and situation model levels (Martínez-Flores et al., 2025). These findings suggest the recruitment of distinct neural systems managing CL and point to the possibility that individual differences in brain structure may significantly contribute to variability in CL and reading comprehension outcomes (van den Broek et al., 2015).

Previous research has linked interindividual differences in brain anatomy, particularly in the inferior frontal gyrus and hippocampus (Keller et al., 2024; Skoe et al., 2017), with variations in reading comprehension and pupillary responses, raising the possibility that neuroanatomical factors shape cognitive benefits derived from acute physical activity. These observations align with growing evidence stressing the importance of individual differences in brain morphology when examining reading comprehension, especially during sensitive developmental stages such as adolescence (Eckert et al., 2016; Perdue et al., 2020; Marks et al., 2024).

This research addresses two gaps. First, although pupil dilation is a well-established biomarker of CL (van der Wel and van Steenbergen, 2018), little is known about how physical activity affects this relationship when individual brain structure is considered (Zou et al., 2023). Second, the majority of volumetric brain research has been conducted outside Latin America, leaving a significant geographical gap (Wassenaar et al., 2020).

Therefore, this study aimed to determine how gray matter volume in specific brain regions moderates the effect of physical activity modality on cognitive load, indexed by pupil dilation, and explore its association with reading comprehension.

Four hypotheses were formulated. H1: C-HIIT would produce superior reading comprehension and lower CL compared to both MICT and the sedentary condition, consistent with the association between high-intensity interval exercise and greater noradrenergic activation. H2: MICT would outperform the sedentary condition on both outcomes, though to a lesser degree than C-HIIT, reflecting a dose-dependent exercise–cognition relationship. H3: Baseline gray matter volume would not independently predict reading comprehension or CL, since structural differences require physiological activation to translate into functional advantages. H4: Gray matter volume would significantly moderate the effect of physical activity modality on cognitive load, tested through condition × volume interaction models, with the strongest modulation expected under C-HIIT. For reading comprehension, an analogous association between individual differences in volume and physical activity effects was expected on the same theoretical grounds; however, since the moderator only varies at the participant level, this could not be formally tested as a moderation effect and was instead examined as a descriptive, exploratory association between individual changes and regional volume. Region-specific expectations were derived from the construction-integration model and existing neuroimaging literature: brainstem and pars opercularis volumes were expected to relate to foundational text processing, whereas hippocampal volume has been associated with higher-order comprehension and contextual integration (Kintsch, 1988; Keller et al., 2024). As no prior study directly examined this interaction, these region-level expectations were treated as exploratory rather than confirmatory.

## 2 Methods

This is a secondary analysis of data obtained from the cross-sectional Cogni-Action Project, conducted between March 2017 and October 2019. Comprehensive methodological details have been previously published in two independent studies (Martínez-Flores et al., 2025; Solis-Urra et al., 2019). This study was registered in the ClinicalTrials.gov database (identifier: NCT03894241) and approved by the Ethics Committee of the authors’ institution. All phases were conducted in accordance with the Declaration of Helsinki.

### 2.1 Participants

From the original sample (*n* = 1,296), two subsamples were identified: 76 participants who underwent MRI neuroimaging (Solis-Urra et al., 2019) and 32 participants who completed an eye-tracking crossover trial (Martínez-Flores et al., 2025). The present study only includes participants for whom both MRI and eye-tracking data were available. Of the 32 crossover participants, 13 had usable MRI data, yielding a final sample of *n* = 13. This reflects data availability rather than *a priori* power calculations; original sample sizes and power justifications are reported in their respective studies. Because gray matter volume, the moderator of central interest, varies only at the participant level, *n* = 13 represents the effective sample size for every condition × volume effect reported in this study, and the study is accordingly framed as an exploratory secondary analysis carrying substantial uncertainty. The crossover design provided 39 participant-condition combinations and 9,556 item-level pupillometry data points nested within participants and texts (Pontifex et al., 2019). *Post-hoc* power analysis (SIMR package (Green and MacLeod, 2016), 1,000 datasets) confirmed power exceeded 99% for cognitive-load models at *n* = 13 and remained ≥80% at *n* ≥ 9. Full details are in the Supplementary material.

We included only right-handed male adolescents enrolled in seventh or eighth grade (12–13 years old) at a subsidized school of middle socioeconomic status, ensuring hemispheric homogeneity (Solis-Urra et al., 2019). The inclusion criteria are as follows: native Spanish speakers with normal or corrected-to-normal vision and no physical, psychiatric, or psychological disabilities. Previous studies report full eligibility details (Martínez-Flores et al., 2025).

### 2.2 Experimental conditions and procedures

Participants were involved in three randomized experimental conditions, each separated by a 2-week washout period, all under supervision in the IRyS laboratory. The order of the three conditions was randomized for each participant using a web-based random sequence generator.^1^ This process was carried out by a recruitment staff member who was not involved in the outcome assessment, consistent with the randomization procedure described in our previously published protocol for this cohort (Martínez-Flores et al., 2025). The three reading texts were randomized independently of the condition order and matched for syntactic complexity and length (ranging from 506 to 555 words). This ensured that text-specific learning would not be confounded by any particular condition or sequence position. All sessions were held on the same weekday and at the same time of day for each participant to minimize circadian and school-schedule confounding effects. In the sedentary condition (SC), adolescents watched a nature documentary for 90 min indoors (20–22 °C, standardized lighting). In the moderate-intensity continuous training (MICT) condition, participants engaged in outdoor self-paced brisk walking targeting ~60% of estimated maximum heart rate (HRmax) (Butte et al., 2018). In the cooperative high-intensity interval training (C-HIIT) condition, participants performed four sets of four cooperative exercises (20:40-s work-to-rest ratio) targeting ≥85% of estimated HRmax (Chen et al., 2014).

All physical activity sessions included a 4-min warm-up and cool-down period. Heart rates were monitored using Polar H10 monitors (HRmax = 220 minus age). Energy expenditure was matched across conditions using basal metabolic rate (BMR) estimates and metabolic equivalent of task (MET) values (SC: 1.3, MICT: 4.9, C-HIIT: 7.4) (Butte et al., 2018). Because higher-intensity protocols require less time to reach equivalent caloric expenditure, the duration of MICT and C-HIIT sessions was shorter than the 90-min SC; this variation in duration was an intended choice to prioritize equivalence in energy expenditure rather than equal session lengths across conditions.

Reading evaluations occurred ~20 min post-intervention. The CONSORT flow chart in Figure 1 presents the study design. A full description of experimental procedures can be found in the Supplementary material.

**Figure 1:**
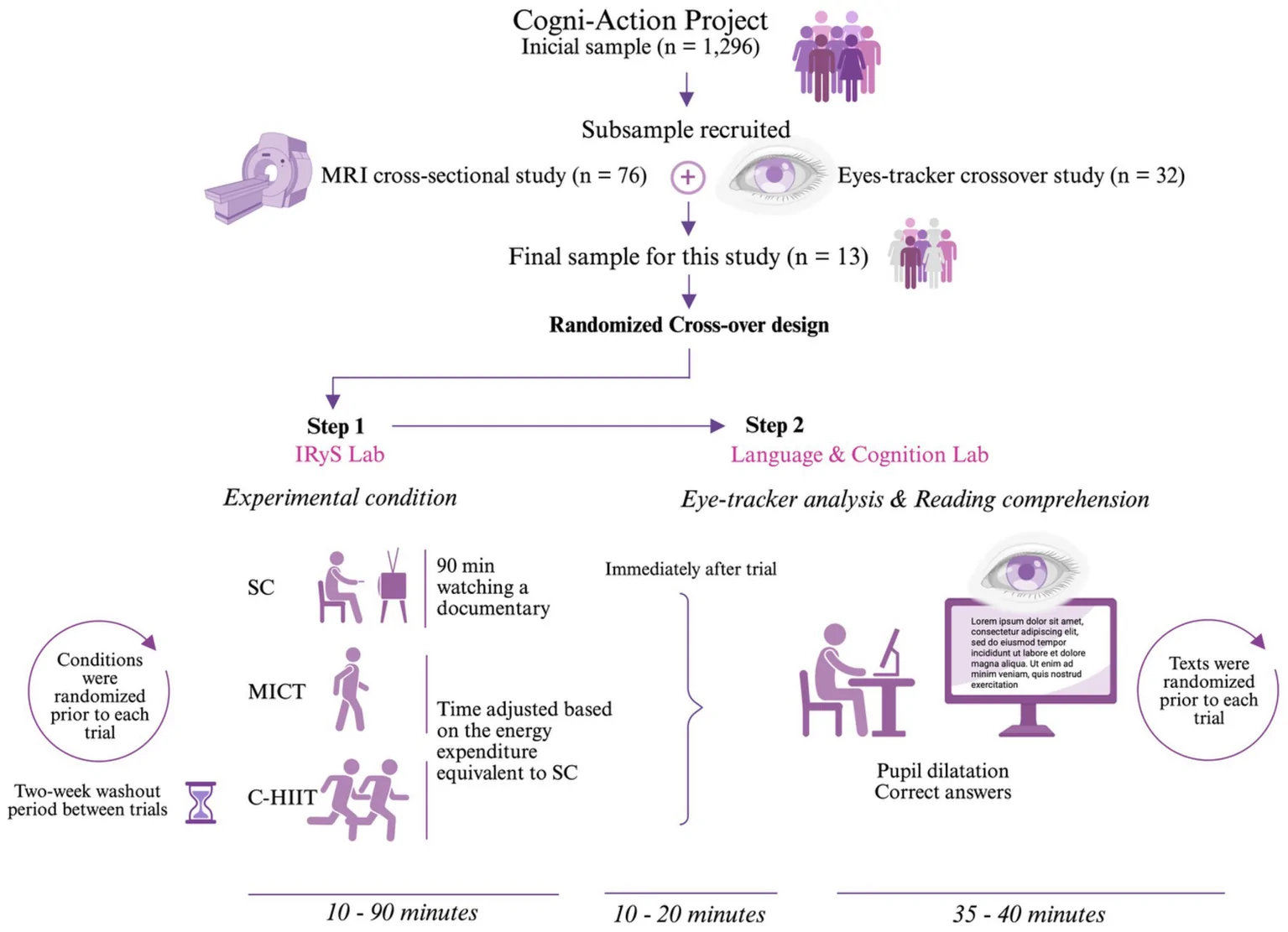
CONSORT-based flowchart of the study design and procedures. SC, sedentary condition; MICT, moderate-intensity continuous training; C-HIIT, cooperative high-intensity interval training.

### 2.3 Cognitive load and comprehension measurements

Participants read three randomized narrative texts in Spanish (Greek and African myths) with similar syntactic complexity and word counts (506–555 words). Pupil dilation during reading served as a physiological proxy for CL (Zou et al., 2023; Engelhardt et al., 2010), and this was captured using a Tobii TX-300 eye tracker (300 Hz sampling rate, monocular tracking), under identical lighting conditions (Lux: 698.67 ± 106.9) (see Figure 2). The pupil dilation data were averaged per area of interest (i.e., individual words) using Tobii Pro Lab software (Tobii AB, Stockholm, Sweden), which performed calibration validation, blink detection, and preprocessing as part of its standard procedure. Participants were medication-free and instructed to abstain from caffeine for 24 h and to obtain ≥8 h of sleep before testing. No trials, participants, or comprehension items were excluded from the analyses; fixations falling outside the text area of interest were removed prior to averaging the pupil data, consistent with standard practice in word-by-word reading studies.

**Figure 2:**
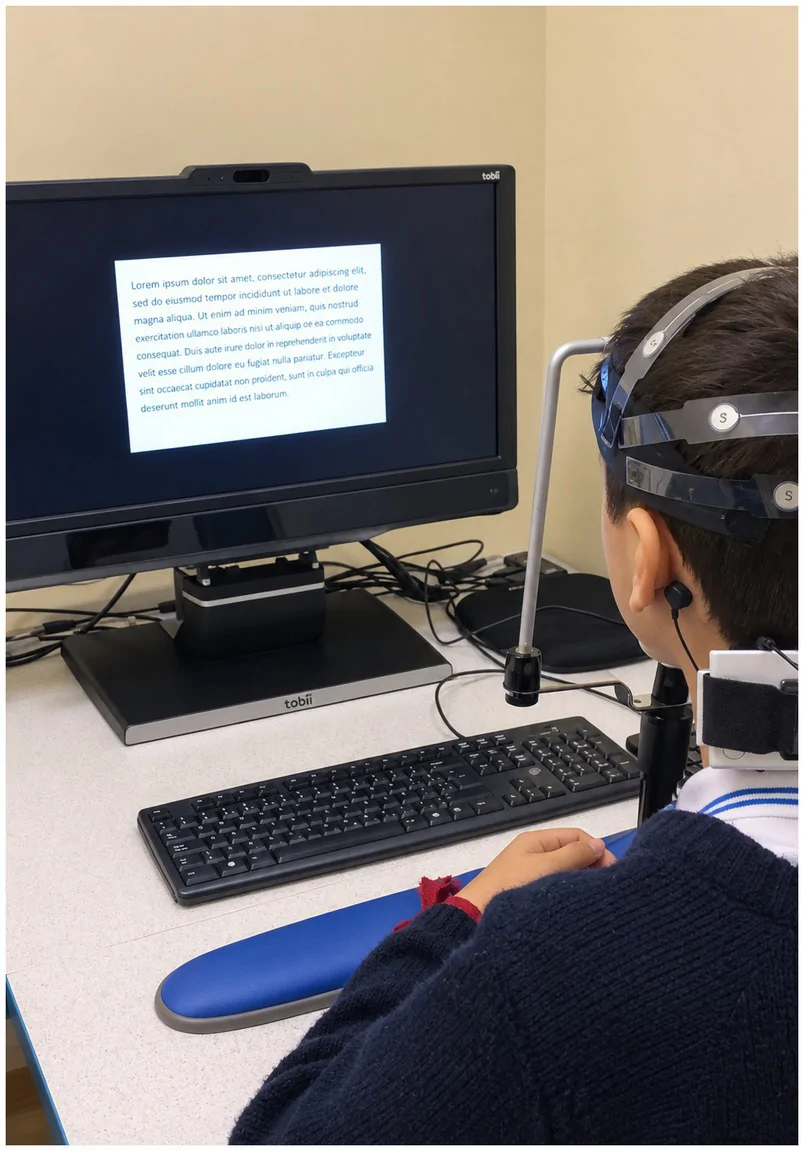
Setup of the reading task with head stabilization for data recording. To ensure full subject anonymity and comply with strict ethical guidelines, the visible human figure has been computer-generated instead of using insufficient methods such as pixelation.

Comprehension was assessed based on seven multiple-choice questions per text, which were validated by three specialists (Fleiss’ Kappa = 0.83). Internal consistency was acceptable (Cronbach’s *α* = 0.76) based on pilot testing. More details can be found in the Supplementary material.

### 2.4 MRI acquisition and processing

T1-weighted MPRAGE images were processed using FreeSurfer 7.4.1 on Neurodesk via the cross-sectional recon-all pipeline. Gray-matter volumes were extracted for the left and right hippocampi, the left pars opercularis, and the brainstem using the Desikan–Killiany atlas (aseg.stats and lh.aparc.stats files). These regions were selected based on three convergent criteria: (1) their established roles as strong neuroanatomical predictors of reading comprehension (Keller et al., 2024; Skoe et al., 2017), (2) their documented sensitivity to acute physical activity through noradrenergic pathways (Tsetsenis et al., 2022; Plini et al., 2023; Yebra et al., 2019), and (3) their functional specialization within the construction-integration reading model. Specifically, the pars opercularis is involved in phonological-orthographic and semantic processing, the hippocampus facilitates contextual integration, and the brainstem regulates arousal, attention, and pupillary responses (Kintsch, 1988; Keller et al., 2024; Basinger and Hogg, 2025). Quality control followed the ENIGMA Consortium Cortical QC Protocol 2.0, including the visual inspection of segmentations, outlier detection (>2.5 SD), motion artifact assessment, and anatomical plausibility verification.

### 2.5 Statistical analysis

All continuous variables were standardized to *z*-scores prior to model fitting. Data normality was assessed using Shapiro–Wilk tests and Q–Q plots. To account for the repeated-measures crossover structure, cognitive load (pupil dilation) was treated as the primary outcome and analyzed using linear mixed-effects models (lmerTest package, R 4.0.1). The experimental condition was entered as a fixed effect, while participant and text were specified as crossed random intercepts. Due to the sample size (*n* = 13), random slopes were not included in the analysis (Barr et al., 2013). For each brain region, the cognitive load model was specified in R (lme4 syntax) as follows:

Pupil dilation~Condition×Volume+(1∣Participant)+(1∣Text)

where Condition is the three-level experimental condition factor (SC, MICT, or C-HIIT), Volume is the standardized gray matter volume of the corresponding region, and Participant and Text are entered as crossed random intercepts.

Effect moderation by gray matter volume was examined using a condition × brain volume interaction term, treating volume as a continuous moderator. This interaction coefficient is reported as the volume-dependent slope difference, which represents the change in the effect of one condition relative to another (with the reference condition for a given contrast) per standard-deviation increase in regional gray matter volume.

Reading comprehension was treated as a secondary, exploratory outcome, given that the moderator of interest (gray matter volume) varies only at the participant level and that a model-based interaction approach would substantially overstate the effective precision available from this sample. For each pairwise condition contrast, within-subject change was summarized as a paired Hedges’ *g* effect size with small-sample correction, with 95% confidence intervals obtained through bootstrap resampling (5,000 resamples; boot package) (Efron and Tibshirani, 1994). An exploratory, uncorrected sign-flip permutation *p*-value was additionally computed for each contrast by randomly reversing the sign of individual differences across 5,000 iterations.

To characterize the individual response patterns provided by the crossover design, a distribution-based threshold was used (Norman et al., 2003). Participants were classified for each contrast in three categories: clear responders (score change at or above one pooled standard deviation of the two compared conditions), participants showing a minor change (improvement below that threshold), or participants showing no change or a decrease in scores. The pooled standard deviation was calculated from the two conditions for each contrast, using the complete cases available for that contrast. This distribution-based approach was preferred over a standardized effect size such as Cohen’s *d* at the individual level because reading comprehension was measured on a discrete, bounded scale (0–7 correct answers), where a change of one point already represents a meaningful step, and standardized individual-level effect sizes are difficult to interpret.

The association between the magnitude of individual score change and regional gray matter volume was explored using Spearman’s correlations, with 95% confidence intervals derived from percentile bootstrap resampling (5,000 resamples). These comprehension analyses are reported descriptively, without a formal significance framework or correction for multiple comparisons across regions, and are interpreted as hypothesis-generating rather than confirmatory.

Statistical inference was based on a within-subject condition permutation procedure: condition labels were randomly shuffled for each participant across 5,000 iterations, preserving the crossover dependency structure (Winkler et al., 2014). To control for family-wise error across all pairwise contrasts and all four brain-region models simultaneously, a max-*T* correction was applied to each permutation run. Ninety-five percent confidence intervals were derived from bootstrap resampling (with 5,000 iterations). The results are reported as standardized *β* coefficients with 95% CIs for linear mixed models (LMMs).

No additional covariates were included, for three reasons: the within-subjects crossover design controls for stable between-participant characteristics (including baseline fitness) via the participant random intercept; condition-level variables such as testing interval, session order, and text assignment were experimentally standardized rather than statistically adjusted; and given *n* = 13, additional covariates would risk overfitting and convergence instability without improving causal inference. Full justification is provided in Supplementary material S.4.

## 3 Results

Descriptive baseline values are presented in Table 1. None of the participant was at risk of excessive abdominal fat or obesity.

**Table 1:** Participants characteristics.

Indicator	Participants mean ± SD	Min–Max range
Age (years)	12.2 ± 0.3	12–13
Body weight (kg)	54.6 ± 12.1	36.5–82.9
Body height (cm)	156 ± 10.3	137–169
BMI (SD)	1.37 ± 1.01	−0.5 to 2.88
Waist-to-height ratio	0.45 ± 0.05	0.40–0.55
Correct answers SC	3.67 ± 1.23	1–5
Correct answers MICT	4.33 ± 1.07	2–6
Correct answers C-HIIT	5.25 ± 1.06	3–7
Left hippocampus volume (mm^3^)	3,898 ± 527	3,015–4,638
Right hippocampus volume (mm^3^)	4,092 ± 408	3,645–4,868
Left pars opercularis volume (mm^3^)	5,807 ± 877	4,118–7,291
Brainstem (mm^3^)	20,516 ± 1,269	18,138–22,538

kg, kilograms; cm, centimeters; SD, standard deviation; mm^3^, cubic millimeters.

Linear mixed-effects models for pupil diameter yielded conditional *R*^2^ values of 0.80–0.81 and ICC values of 0.80, indicating that between-participant and between-text clustering accounted for the majority of the variance in pupil diameter. Binomial mixed-effects models for reading comprehension yielded conditional *R*^2^ values of 0.44–0.56 and ICC values of 0.37–0.43, indicating adequate random structure partitioning across participants and texts. Physical activity modality significantly predicted pupil diameter and reading comprehension across all models (all *p* < 0.001).

### 3.1 Main effects of physical activity modality

MICT was associated with a higher cognitive load than SC across all four brain-region models (*β* range: 0.12–0.23; all *p* = <0.001). In addition, MICT resulted in a lower cognitive load than C-HIIT across all models (*β* range: −0.17 to −0.19; all *p* = <0.001). The direction of the C-HIIT vs. SC comparison was region-dependent: differences were non-significant in both hippocampal models (*β* ≈ −0.03; both *p* > 0.20); C-HIIT generated lower cognitive load than SC in the pars opercularis model [*β* = −0.07, 95% CI (−0.11, −0.03), *p* = <0.001]; and C-HIIT generated modestly higher cognitive load than SC in the brainstem model [*β* = 0.05, 95% CI (0.01, 0.09), *p* = 0.031]. Reading comprehension scores were higher under both active conditions than under SC, with the largest difference for C-HIIT.

### 3.2 Volume × condition interactions: cognitive load

Gray matter volume moderated the effect of physical activity modality on cognitive load, with directionality that varied systematically by brain region. In hippocampal models, larger volume was associated with steeper cognitive load increases for active conditions compared to SC, with C-HIIT showing a markedly stronger effect than MICT. For the left hippocampus, the volume-dependent slope difference relative to SC was as follows: *β* = 0.26 [95% CI (0.21, 0.30), *p* = <0.001] for C-HIIT and *β* = 0.06 [95% CI (0.02, 0.11), *p* = 0.004] for MICT; C-HIIT generated a steeper slope than MICT by *β* = 0.19 [95% CI (0.17, 0.22), *p* = <0.001]. The right hippocampus showed a comparable pattern for C-HIIT [*β* = 0.15, 95% CI (0.11, 0.18), *p* = <0.001], while MICT did not show significant modulation (*β* = −0.002, *p* = 0.995).

In pars opercularis and brainstem models, the direction was reversed: larger volumes were associated with reduced cognitive load during active conditions compared to SC. For the pars opercularis, the volume-dependent slope difference relative to SC was *β* = −0.30 [95% CI (−0.34, −0.26), *p* = <0.001] for C-HIIT and *β* = −0.17 [95% CI (−0.21, −0.14), *p* = <0.001] for MICT, with C-HIIT showing significantly steeper attenuation [*β* = −0.13, 95% CI (−0.16, −0.10), *p* = <0.001]. For the brainstem, the attenuation was comparable for both active conditions [C-HIIT: *β* = −0.32, 95% CI (−0.36, −0.28); MICT: *β* = −0.29, 95% CI (−0.33, −0.26); both *p* = <0.001], with no significant difference between them (*β* = −0.02, *p* = 0.232). Across all four models, C-HIIT maintained lower absolute cognitive load than MICT, regardless of the region. Figure 3 shows forest plots for all contrasts.

**Figure 3:**
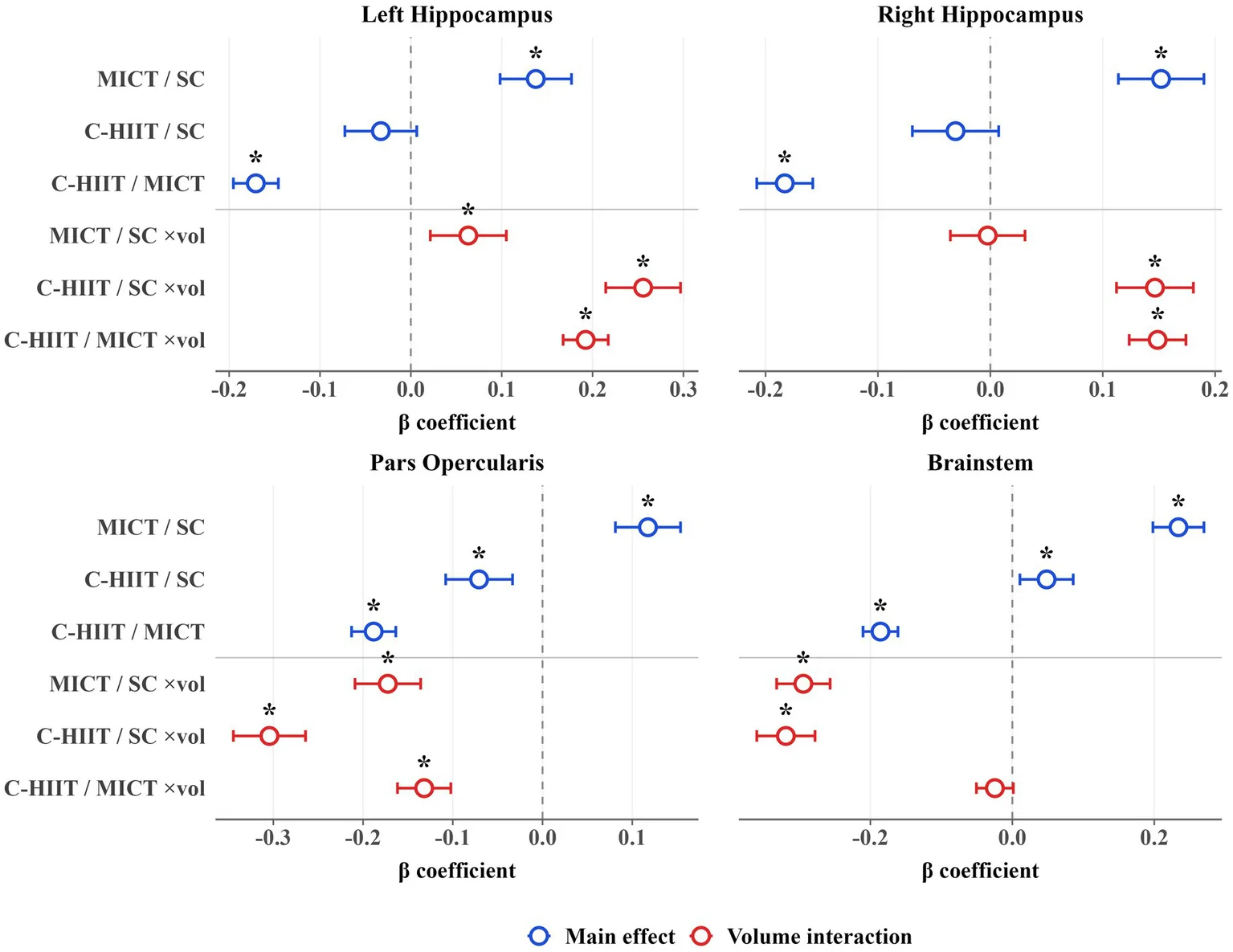
Brain volume moderates physical activity effects on cognitive load. Data are presented as a comparator vs. reference. MICT, moderate-intensity continuous training; SC, sedentary condition; C-HIIT, cooperative high-intensity interval training. ^*^<0.05.

To further characterize these interactions, condition contrasts were additionally estimated at low, mean, and high regional gray matter volume, using the same bootstrap resampling and permutation-based max-*T* correction described in Section 2.5. This decomposition in Table 2 revealed that the direction of the C-HIIT vs. SC contrast reversed across the volume range in both hippocampal regions, whereas in the pars opercularis and brainstem, the reversal occurred in the opposite direction. Every contrast reaching significance under permutation-based max-*T* correction was also supported by a bootstrap confidence interval excluding zero, indicating convergent evidence across two independent inferential approaches despite the modest sample size. Conversely, a small number of contrasts for which the bootstrap interval excluded zero did not reach significance once the family-wise max-*T* correction was applied, indicating that the correction was acting conservatively rather than simply ratifying findings that would already have appeared significant under an uncorrected approach.

**Table 2:** Condition contrasts on cognitive load at low, mean, and high regional gray matter volume.

Region	Volume level	Contrast	*β*	95% CI	*p* (max-*T*)
Left hippocampus	Low	MICT vs. SC	0.074	[−0.004, 0.144]	0.248
		C-HIIT vs. SC	−0.289	[−0.356, −0.224]	**<0.001**
		C-HIIT vs. MICT	−0.363	[−0.397, −0.330]	**<0.001**
	Mean	MICT vs. SC	0.138	[0.098, 0.176]	**<0.001**
		C-HIIT vs. SC	−0.033	[−0.073, 0.007]	0.557
		C-HIIT vs. MICT	−0.171	[−0.196, −0.146]	**<0.001**
	High	MICT vs. SC	0.201	[0.160, 0.243]	**<0.001**
		C-HIIT vs. SC	0.223	[0.176, 0.270]	**<0.001**
		C-HIIT vs. MICT	0.022	[−0.015, 0.059]	0.870
Right hippocampus	Low	MICT vs. SC	0.154	[0.095, 0.213]	**<0.001**
		C-HIIT vs. SC	−0.177	[−0.237, −0.120]	**<0.001**
		C-HIIT vs. MICT	−0.332	[−0.367, −0.297]	**<0.001**
	Mean	MICT vs. SC	0.152	[0.114, 0.189]	**<0.001**
		C-HIIT vs. SC	−0.031	[−0.069, 0.007]	0.597
		C-HIIT vs. MICT	−0.183	[−0.208, −0.158]	**<0.001**
	High	MICT vs. SC	0.150	[0.110, 0.190]	**<0.001**
		C-HIIT vs. SC	0.115	[0.071, 0.160]	**<0.001**
		C-HIIT vs. MICT	−0.034	[−0.072, 0.002]	0.431
Pars opercularis	Low	MICT vs. SC	0.290	[0.239, 0.339]	**<0.001**
		C-HIIT vs. SC	0.234	[0.184, 0.284]	**<0.001**
		C-HIIT vs. MICT	−0.056	[−0.095, −0.017]	**0.043**
	Mean	MICT vs. SC	0.117	[0.082, 0.153]	**<0.001**
		C-HIIT vs. SC	−0.071	[−0.106, −0.033]	**0.003**
		C-HIIT vs. MICT	−0.188	[−0.213, −0.163]	**<0.001**
	High	MICT vs. SC	−0.055	[−0.106, −0.004]	0.278
		C-HIIT vs. SC	−0.375	[−0.431, −0.315]	**<0.001**
		C-HIIT vs. MICT	−0.320	[−0.358, −0.281]	**<0.001**
Brain stem	Low	MICT vs. SC	0.529	[0.471, 0.584]	**<0.001**
		C-HIIT vs. SC	0.367	[0.306, 0.427]	**<0.001**
		C-HIIT vs. MICT	−0.161	[−0.197, −0.126]	**<0.001**
	Mean	MICT vs. SC	0.234	[0.199, 0.270]	**<0.001**
		C-HIIT vs. SC	0.048	[0.011, 0.086]	0.097
		C-HIIT vs. MICT	−0.186	[−0.210, −0.161]	**<0.001**
	High	MICT vs. SC	−0.060	[−0.106, −0.013]	0.087
		C-HIIT vs. SC	−0.271	[−0.320, −0.219]	**<0.001**
		C-HIIT vs. MICT	−0.210	[−0.246, −0.175]	**<0.001**

Data are presented as comparator vs. reference. We obtained confidence intervals via parametric bootstrap resampling (5,000 resamples per model). Bold rows indicate contrasts significant at *p* < 0.05 under a within-subject permutation procedure (5,000 iterations) with max-*T* correction for family-wise error across all 36 contrasts. SC, sedentary condition; MICT, moderate-intensity continuous training; C-HIIT, cooperative high-intensity interval training.

### 3.3 Volume and condition associations with reading comprehension (exploratory)

Given that gray matter volume varies only at the participant level (*n* = 13), reading comprehension was analyzed descriptively as a secondary, hypothesis-generating outcome rather than using a model-based interaction approach.

Paired comparisons showed higher reading comprehension scores in C-HIIT relative to SC [Hedges’ *g* = 0.87, 95% CI (0.49, 1.49), exploratory permutation *p* = 0.007], with smaller and less consistent differences for MICT relative to SC [*g* = 0.55, 95% CI (0.06, 2.11), *p* = 0.088] and for C-HIIT relative to MICT [*g* = 0.50, 95% CI (0.00, 1.20), *p* = 0.068] (Figure 4).

**Figure 4:**
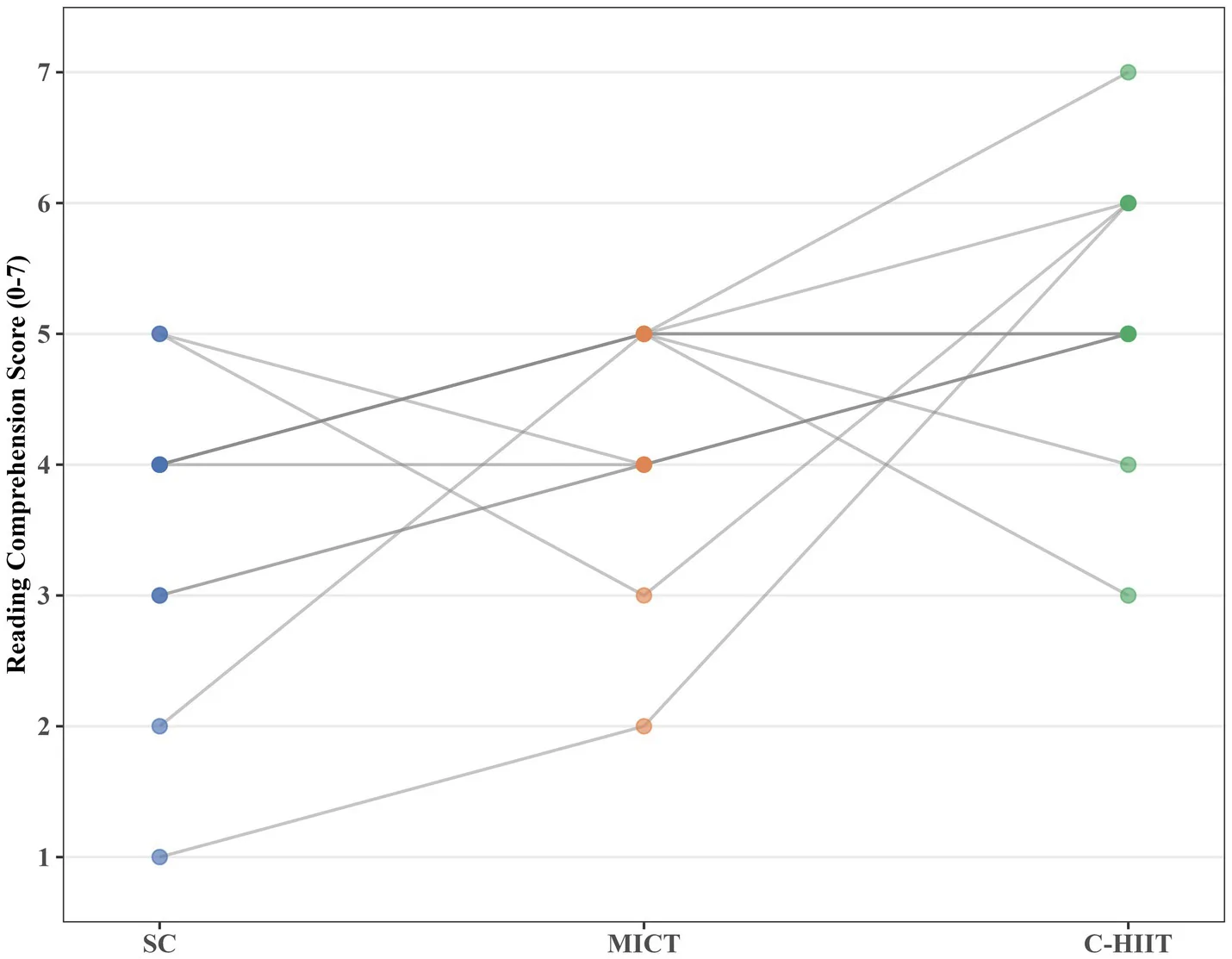
Individual trajectories of reading comprehension scores across experimental conditions. Each line represents one participant (*n* = 13). Points are jittered by condition; correct answers range from 0 to 7. SC, sedentary condition. MICT, moderate-intensity continuous training; C-HIIT, cooperative high-intensity interval training.

Classifying participants by within-subject change relative to a threshold of one pooled standard deviation, clear responders were most common from MICT to C-HIIT (8 of 13 participants, 61.5%) and from SC to C-HIIT (5 of 13, 38.5%), whereas from SC to MICT most participants showed a smaller improvement below the responder threshold rather than a clear response (9 of 13, 69.2%) (Figure 5).

**Figure 5:**
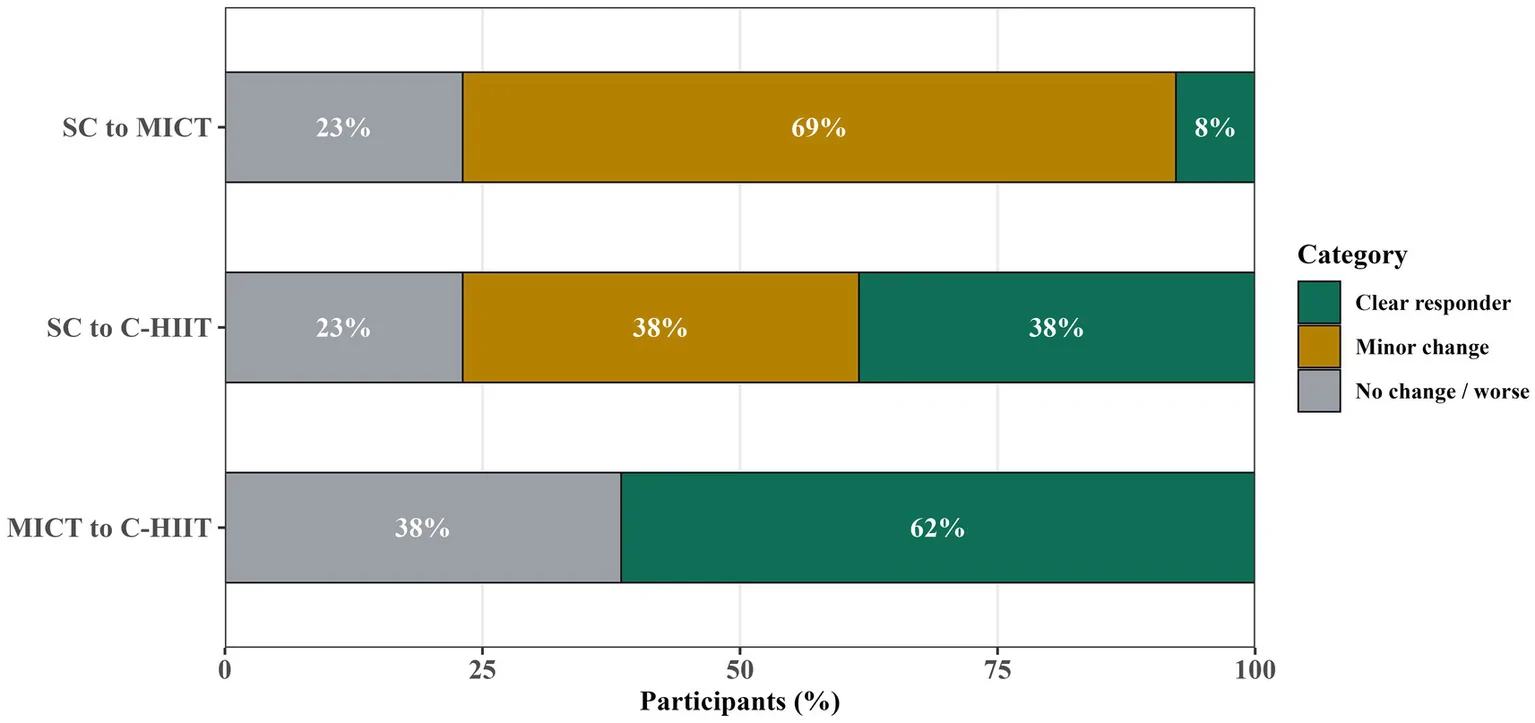
Proportion of participants classified as responders in reading comprehension for each pairwise condition contrast. Participants were classified as clear responders (change at or above one pooled standard deviation of the two compared conditions), showing a minor change (improvement below that threshold), or showing no change or decrease. SC, sedentary condition; MICT, moderate-intensity continuous training; C-HIIT, cooperative high-intensity interval training.

The magnitude of individual improvement in reading comprehension was associated with pars opercularis volume for both the SC to C-HIIT contrast [Spearman rho = 0.53, 95% CI (0.09, 0.77)] and the MICT to C-HIIT contrast [rho = 0.59, 95% CI (0.13, 0.84)], indicating that participants with greater pars opercularis volume tended to show larger comprehension gains under C-HIIT. No other region and contrast combination showed a confidence interval excluding zero (hippocampal volumes: rho range −0.24 to 0.15; brainstem: rho range −0.35 to 0.42) (Figure 6).

**Figure 6:**
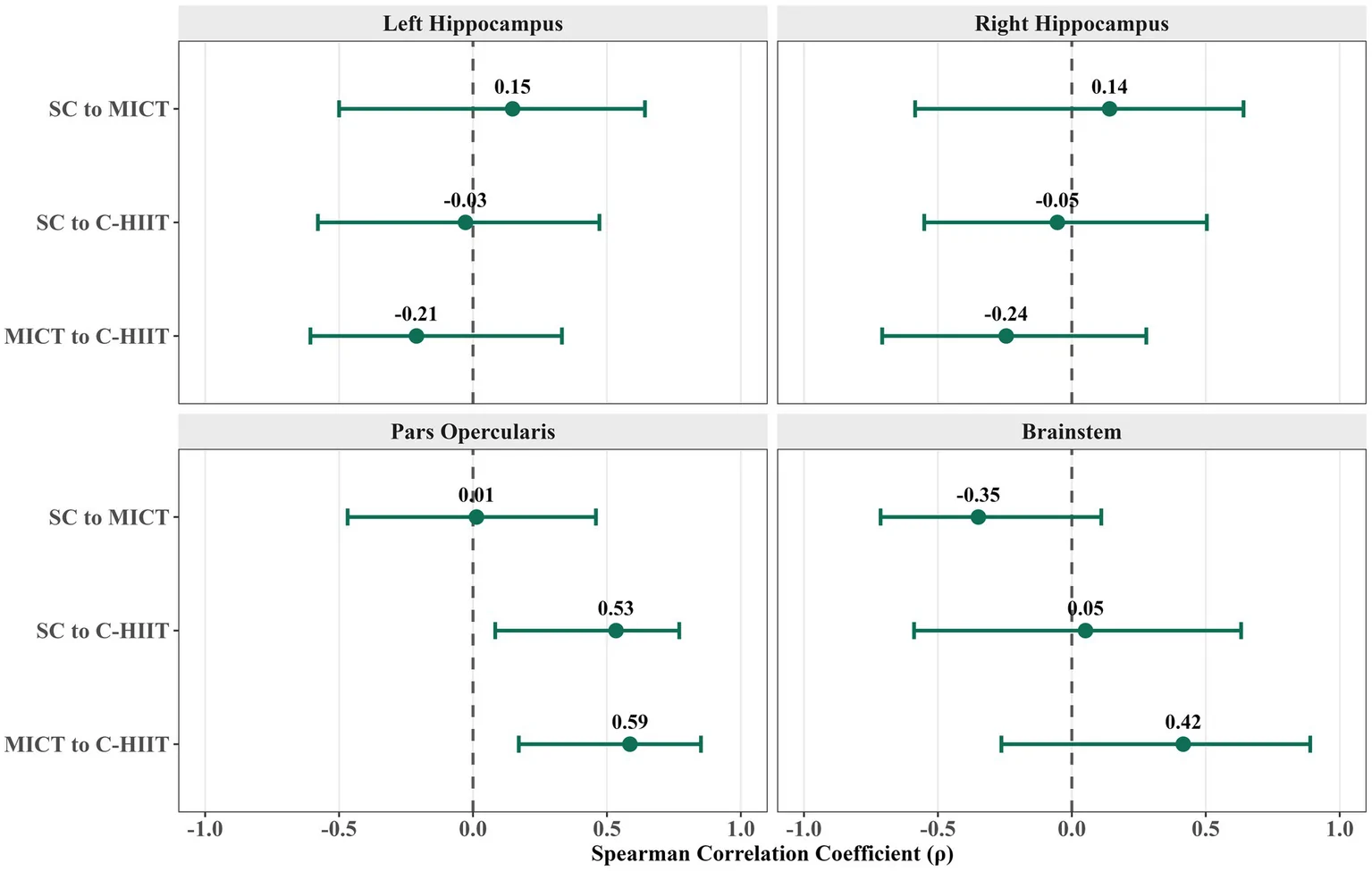
Exploratory association between individual change in reading comprehension and regional gray matter volume. Points represent Spearman’s correlation coefficients between the within-subject change in reading comprehension score and regional gray matter volume; bars represent 95% confidence intervals obtained through percentile bootstrap resampling. Each panel corresponds to a brain region, and each row within a panel corresponds to a pairwise condition contrast. SC, sedentary condition; MICT, moderate-intensity continuous training; C-HIIT, cooperative high-intensity interval training.

## 4 Discussion

Regional gray matter volume systematically moderated the effect of acute physical activity modality on cognitive load in adolescents, with the direction and magnitude of this moderation varying consistently across all four brain regions examined. C-HIIT was associated with lower cognitive load and higher reading comprehension compared to both SC and MICT. For cognitive load, both advantages were amplified by volume interactions in a model-based framework. For reading comprehension, exploratory analyses further suggested that participants with greater pars opercularis volume tended to show larger individual gains under C-HIIT, although this association was descriptive and limited to a single region. Crucially, gray matter volume did not independently predict cognitive load in any model, indicating that neuroanatomical capacity requires appropriate physiological activation to manifest functionally. For reading comprehension, volume was examined only via individual change scores, not as a comprehension predictor.

### 4.1 Physical activity effect on reading comprehension and CL

C-HIIT was the only condition to show a consistent advantage over both SC and MICT, combining lower cognitive load relative to MICT with higher reading comprehension scores, a pattern consistent with more efficient processing, though the mechanisms underlying this efficiency cannot be directly determined from the present data. This pattern was reflected in pupil dilation indicating differential CL levels in real time during reading, alongside comprehension scores that were consistently higher under C-HIIT across the pairwise contrasts examined, reflecting distinct patterns of reading processing depending on the specific brain structures examined.

The construction-integration model postulates that comprehension involves: (1) surface code representation, (2) textbase (propositions and explicit relationships), and (3) the situation model (contextual and relational representation), progressing from less to more complex processes (Kintsch, 1988). The pars opercularis and brainstem may be involved in the surface code and textbase processes, while the hippocampus intervenes in the situation model, all strong predictors of reading comprehension (Keller et al., 2024).

The pars opercularis is a key node in phonological-orthographic processing and subvocal recoding, with a critical role in language and semantic processing (Keller et al., 2024; Glezer et al., 2016). The brainstem, particularly the locus coeruleus, is key to top-down attentional processes that support initial information processing (Katsuki and Constantinidis, 2014). The hippocampus is related to information integration based on prior knowledge and contextual information (Yebra et al., 2019; Hausser et al., 2021). Acute physical activity, particularly C-HIIT, appears linked to enhanced noradrenergic signaling: locus coeruleus excitability increases, generating brain catecholamines (McMorris, 2021) that project to the prefrontal cortex, inferior frontal gyrus (including pars opercularis), and hippocampus, improving processing speed, information integration, and inhibitory control (Tsetsenis et al., 2022; Hansen et al., 2024). This response may also modulate theta and gamma oscillations to synchronize activity between brain regions (Tully et al., 2007; Guedj et al., 2017), though these mechanisms remain speculative.

C-HIIT was associated with lower pupil dilation (reduced CL) while maintaining higher comprehension scores, suggesting enhanced comprehension with lower “cognitive cost.” This pattern is consistent with more efficient resource allocation in less complex reading stages (surface code and textbase): the pars opercularis, one of the strongest predictors of reading comprehension due to its role in semantic and language processing (Keller et al., 2024), and the brainstem, through sustained attentional support (Hansen et al., 2024), may facilitate better coupling between foundational stages and hippocampal activity supporting the situation model, preserving resources for the more complex integration processes that demand higher CL (Keller et al., 2024). These varied patterns across regions reflect functional specialization: brainstem and pars opercularis showed volume-dependent CL reductions under C-HIIT, and pars opercularis volume was also associated, in exploratory analyses, with the individual magnitude of comprehension gains under C-HIIT relative to both SC and MICT, whereas hippocampal and brainstem volume showed no such association. Critically, baseline volume predicted cognitive load only when interacting with physical activity in a formal model, suggesting neuroanatomical capacity requires appropriate physiological activation to manifest functionally; the comprehension-volume association should be interpreted with the same logic, though it rests on a correlational, hypothesis-generating analysis rather than a fitted interaction. C-HIIT’s superior outcomes are consistent with greater locus coeruleus activation and phasic noradrenergic bursts (Vazey et al., 2018), whereas MICT’s sustained moderate intensity may be insufficient to fully engage these pathways. These findings extend those of Martínez-Flores et al. (Martínez-Flores et al., 2025) by suggesting that the differential effects of C-HIIT and MICT on reading comprehension and cognitive load are not uniform across individuals and that at least part of this variability may relate to baseline regional brain morphology, a moderating dimension unavailable in the original study and one that future, adequately powered studies should examine more formally.

Beyond brain morphology, chronological age and developmental stage represent another individual-difference dimension shown to moderate exercise-cognition relationships in school-age populations. For instance, for primary school children, the positive association between physical strength and short-term memory was significant only among children above the sample’s mean age, with no reliable association detected among younger children (Agostino et al., 2023). This broader literature reinforces that individual neurobiological and developmental characteristics, rather than physical activity modality alone, jointly shape the cognitive outcomes of acute exercise, consistent with the region-specific, volume-dependent patterns reported here.

Regarding the C-HIIT vs. MICT difference, one possible additional mechanism, not measured in the present study, involves cerebral lactate metabolism: the cyclical nature of C-HIIT may generate repeated homeostatic perturbations enhancing lactate shuttling to the brain, and executive function benefits from physical activity have been related to cerebral lactate metabolism (Xue et al., 2022). Higher-intensity exercise may also produce greater brain noradrenergic responses, as animal models show higher-intensity physical activity generates greater brain catecholamine release (Basso and Suzuki, 2016; Arbat-Plana et al., 2019). This response may modulate glutamate, dopamine, and serotonin, key to hippocampal function (Moreno-Castilla et al., 2017; Song and Tan, 2023), potentially modulating glutamate, dopamine, and serotonin release relevant to hippocampal function.

### 4.2 Strengths and limitations

The crossover design minimized interindividual variability (Pontifex et al., 2019). To our knowledge, this is the first study to assess how gray matter volume in key reading regions modulates physical activity’s effect on CL and reading comprehension in a Latin American adolescent sample, contributing to a recognized geographical gap in the literature.

Important limitations must be acknowledged. The proposed noradrenergic mechanism was not directly measured, as we did not obtain catecholamine assays or locus coeruleus neuroimaging, so the mechanistic interpretation in Section 4.1 remains inferential. The modest sample size (*n* = 13) limits statistical power and generalizability. We did not formally assess pubertal stage, and residual developmental variability within the narrow age band cannot be ruled out. The male-only sample prevents examining sex differences in brain volume × exercise interactions. C-HIIT and MICT differ in both intensity and modality, preventing disentanglement of these factors. Age-predicted HRmax formulas lack individual precision. Cross-sectional brain volume measurement precludes causal inferences about structural differences. MICT sessions were conducted outdoors while SC and C-HIIT took place indoors; potential confounds from natural light exposure, ambient temperature, and terrain surface cannot be fully excluded. Gray matter volume was extracted at the level of whole anatomical structures (hippocampus, pars opercularis, brainstem) rather than their constituent subfields or substructures, which are known to be functionally heterogeneous; subfield-level segmentation, ideally using dedicated high-resolution sequences and a larger sample, would be a valuable direction for future studies. The association between reading comprehension change and gray matter volume was examined descriptively rather than through a fitted interaction model, given that the moderator varies only at the participant level; these correlations should be considered hypothesis-generating, are not corrected for multiple comparisons across regions, and require replication in a large sample before any causal or clinical interpretation.

## 5 Conclusion

This study provides evidence that gray matter volume in brain regions critical for reading comprehension does not independently predict cognitive load or reading comprehension in school-age adolescents, but instead moderates cognitive load through interaction with physical activity modality, with C-HIIT consistently producing the greatest reduction in cognitive load once brain volume was considered. A parallel, exploratory association between pars opercularis volume and individual gains in reading comprehension under C-HIIT points in the same direction but requires confirmation in a larger sample. These findings highlight the relevance of jointly considering physical activity modality and individual brain morphology when designing interventions to maximize cognitive benefits in school settings.

## Data Availability

The data presented in this study are available on request from the corresponding author. The data are not publicly available due to ethical concerns (carlos.cristi.montero@gmail.com). The complete analysis code used in this study is publicly available on GitHub and has been permanently archived via Zenodo at https://doi.org/10.5281/zenodo.21644144. The repository includes the full R script used to fit all statistical models, perform bootstrap resampling and permutation-based inference, and generate all figures and tables reported in this manuscript, along with a README describing the analysis pipeline and software requirements.

## Footnotes

1
randomizer.org
