# Supplementary material for: Gray matter volume moderates the effect of acute physical activity on cognitive load and relates to reading comprehension in adolescents: the Cogni-Action Project
Source: Front Psychol. 2026 Oct 6;17:1930058. doi: 10.3389/fpsyg.2026.1930058 (PMC13680183; doi:10.3389/fpsyg.2026.1930058)
Supplement: Supplementary file 1 [file Table_1.DOCX]

*S.1. Power analysis (from Section 2.1)*

To characterize the sensitivity of this secondary analysis, we conducted post-hoc simulation-based power analysis, which appropriately accounts for the nested crossover structure and crossed random effects (1). Specifically, we generated 1,000 new datasets by drawing from the estimated parameter distributions of the fitted mixed-effects model and refitting the full model to each simulated dataset with the SIMR r package approach (1). We tested both the strongest and weakest significant C-HIIT × brain volume interactions across model families, providing a conservative bound. Power exceeded 99% across all four models at n = 13, with 100% convergence in all simulations. Power curve analysis (subsampling at n = 5, 7, 9, 11, 13) confirmed that power remained ≥80% at n ≥ 9 in all cases, indicating that the study was adequately powered to detect the interaction effects on which the conclusions rest, even under the most conservative scenario.

*S.2. Experimental conditions and procedures description of within-subject cross-over trial*

Participants completed three experimental conditions with two weeks of wash out periods between trials in a randomized order at the IRyS Laboratory, always under the supervision of a trained research assistant. In the Sedentary Condition (SC), adolescents watched a nature documentary for 90 minutes in a controlled indoor environment emulating at certain point a regular school class (20–22 °C, standardized lighting, and absence of environmental noise). To ensure participants remained attentive, the assistant periodically asked simple content-related questions.

In the Moderate-Intensity Continuous Training (MICT) condition, participants engaged in a self-paced brisk walk outdoors, targeting approximately 60% of their estimated maximum heart rate (2). In the Cooperative High-Intensity Interval Training (C-HIIT) condition, participants performed four sets of four cooperative exercises designed to engage cardiorespiratory, speed-agility, and coordinative demands (3). These activities followed a 20:40-second work-to-rest ratio and aimed to reach at least 85% of estimated HRmax. The term cooperative exercises refers to cognitively and physically demanding tasks that require interpersonal interaction, coordination, and rapid decision-making (3).

All physical activity sessions included a 4-minute warm-up (running, lateral movements, dynamic stretching) and a 4-minute cool-down. Physical activity intensity was monitored using Polar H10 heart rate monitors. Maximum heart rate was calculated using the 220 minus age formula and effort calculated as (exercise HR/[220–age])×100. Although heart rate–based formulas are widely used in children and adolescents, they may lack individual precision; we applied such a formula to ensure comparability with the large body of prior studies in similar populations (4). Nevertheless, this constitutes a limitation of our study, and future research should consider more accurate approaches for estimating exercise intensity (e.g., graded exercise testing) (4).

Both physical activity conditions (MICT and C-HIIT) were performed outdoors to emulate typical school recess environments, whereas the SC condition was carried out indoors to mirror a classroom setting. This indoor–outdoor contrast captures ecologically valid differences in adolescents’ daily activity contexts and, rather than representing a methodological weakness, should be regarded as an important contextual factor to acknowledge when interpreting cognitive outcomes (5,6).

Energy expenditure was matched across all three conditions to ensure comparable physiological demands delivered through different modalities. Specifically, basal metabolic rate (BMR) was estimated for each participant, and energy expenditure during the SC was calculated as METS (1.3) × BMR (kcal/min) × duration (90 minutes). For MICT and C-HIIT, the duration of physical activity was tailored to match this estimated expenditure using their respective MET values (4.9 and 7.4, respectively) (2). For detailed methodology on energy matching procedures, please refer to a previous published study (7).

Each participant attended three sessions, scheduled every two weeks to allow a wash-out period, with session order randomized and held on the same weekday after school. A typical session involved: (1) Arrival at the IRyS Laboratory for anthropometric measurements and randomization into the physical activity protocol (duration: 10–90 minutes); (2) execution of the assigned physical activity intervention; (3) escorted to the "Language & Cognition" Laboratory for a reading evaluation initiated within 20 minutes of physical activity completion (lasting 35–40 minutes); and (4) a comprehension test of seven questions (3–5 minutes) before being escorted back to school. The reading texts were randomized across sessions, such that all participants completed the three physical activity protocols and read the three texts under different conditions. For more information on the experimental protocol, please refer to a previous published study (7).

*S.3. Cognitive Load and Comprehension Measurements*

In the Lenguaje & Cognición Laboratory at the authors' institution, participants read three randomized narrative texts in Spanish (Greek and African myths) designed by linguistic experts. All texts had similar syntactic complexity and word counts (506–555 words) to minimize learning effects.

Previous studies have established that pupil dilation is a reliable physiological indicator of CL during reading, particularly in response to syntactic complexity, lexical difficulty, and discourse integration demands (8,9). In our study, we did not include a separate behavioral task to assess CL; instead, we used pupil dilation during reading as an established proxy for moment-to-moment CL (8,10).

To ensure consistency in pupil measurement, all participants were assessed under identical conditions and lighting environments (Lux mean ± SD of 3 measures 698.67± 106.9). Furthermore, participants were medication-free and were instructed to abstain from caffeine consumption for 24 hours before testing and to obtain a minimum of 8 hours of sleep the night prior to the experimental sessions, according to guidelines for pupilography (11). The texts were carefully designed to correspond with students' curricular knowledge and the subjects they were studying at their educational level, minimizing the potential confounding effects of arousal or luminance-induced pupil changes. Pupil dilatation as proxy of CL was captured using the Tobii TX-300 eye tracker, operating at a sampling rate of 300 Hz with monocular tracking. Stimuli were presented on a screen with a resolution of 1920 × 1080 pixels. Pupil dilation was averaged per area of interest (i.e., individual words) using the Tobii Pro Lab software (Tobii AB, Stockholm, Sweden), which performed calibration validation, blink detection, and preprocessing as part of its standard pipeline. The experiment was constructed, data collected, and areas of interest (i.e., individual words) defined using Tobii Pro Lab software. Reading evaluations began about 20 minutes after each experimental condition, simulating a typical school recess, and comprehension was assessed with seven questions per text. The test consisted of multiple-choice questions with a single correct option, enabling an objective and standardized evaluation process. To validate the content, three specialists in reading instruction and educational assessment were consulted. They examined each item to ensure clarity, relevance, and alignment with the targeted comprehension level. The level of inter-rater agreement was assessed using the Fleiss' Kappa index, yielding a value of 0.83, which reflects a high degree of concordance among the experts. Due to the exploratory nature of the reading analysis, the number of questions was intentionally limited.

To assess the reliability of the reading comprehension questions, a pilot study was conducted with a group in a subsample of students from the original crossover trial sample who shared similar characteristics with the participants included in the final analysis. The responses were analyzed using Cronbach's alpha coefficient, resulting in a value of 0.76. This indicates an acceptable level of internal consistency for a test of this nature, particularly given the limited size of the pilot sample. Text validation procedures, including syntactic complexity criteria and readability assessments, were conducted as part of the material development process. For more detailed information on the eye tracking measurement, including processing of the data, and the text used, please refer to a previous published study(7).

*S.4. Covariates rationale (from Section 2.5)*

No additional covariates were included for three complementary reasons. First, the within-subjects crossover design inherently controls for all stable between-participant characteristics (including baseline fitness, cognitive capacity, and anthropometric variables), as each participant serves as their own control across conditions; these sources of variance are absorbed by the participant random intercept. Second, condition-level variables that could vary between sessions were experimentally standardized: the interval between exercise and cognitive testing was fixed at approximately 20 minutes, session order was randomized, texts were counterbalanced, and testing environment was held constant. Third, model parsimony was prioritized given the sample size: including additional covariates with n = 13 would introduce overfitting and convergence instability without improving causal inference, given that the design already eliminates the confounds they would target.

**References**

1.	Green P, MacLeod CJ. SIMR: an R package for power analysis of generalized linear mixed models by simulation. Methods Ecol Evol. 2016;7(4):493–8. doi:10.1111/2041-210X.12504

2.	Butte NF, Watson KB, Ridley K, Zakeri IF, McMurray RG, Pfeiffer KA, et al. A Youth Compendium of Physical Activities: Activity Codes and Metabolic Intensities. Med Sci Sports Exerc. 2018 Feb;50(2):246–56. doi:10.1249/MSS.0000000000001430 PubMed PMID: 28938248; PubMed Central PMCID: PMC5768467.

3.	Chen AG, Yan J, Yin HC, Pan CY, Chang YK. Effects of acute aerobic exercise on multiple aspects of executive function in preadolescent children. Psychol Sport Exerc. 2014 Nov 1;15(6):627–36. doi:10.1016/j.psychsport.2014.06.004

4.	Cicone ZS, Holmes CJ, Fedewa MV, MacDonald HV, Esco MR. Age-Based Prediction of Maximal Heart Rate in Children and Adolescents: A Systematic Review and Meta-Analysis. Res Q Exerc Sport. 2019 Jul 3;90(3):417–28. doi:10.1080/02701367.2019.1615605 PubMed PMID: 31157608.

5.	Herold F, Müller P, Gronwald T, Müller NG. Dose-Response Matters! - A Perspective on the Exercise Prescription in Exercise-Cognition Research. Front Psychol. 2019;10:2338. doi:10.3389/fpsyg.2019.02338 PubMed PMID: 31736815; PubMed Central PMCID: PMC6839278.

6.	Mavilidi MF, Vazou S, Lubans DR, Robinson K, Woods AJ, Benzing V, et al. How physical activity context relates to cognition across the lifespan: A systematic review and meta-analysis. Psychol Bull. 2025;151(5):544–79. doi:10.1037/bul0000478

7.	Martínez-Flores R, Julio C, Ibáñez R, Campos-Rojas C, Jarpa M, Supèr H, et al. Impact of Physical Activity Modalities on Text Processing and Comprehension in Adolescents. J Exp Educ. 2025;1–16. doi:10.1080/00220973.2025.2513244

8.	Zou L, Herold F, Ludyga S, Kamijo K, Müller NG, Pontifex MB, et al. Look into my eyes: What can eye-based measures tell us about the relationship between physical activity and cognitive performance? J Sport Health Sci. 2023 Sep;12(5):568–91. doi:10.1016/j.jshs.2023.04.003 PubMed PMID: 37148971; PubMed Central PMCID: PMC10466196.

9.	Engelhardt PE, Ferreira F, Patsenko EG. Pupillometry reveals processing load during spoken language comprehension. Q J Exp Psychol 2006. 2010 Apr;63(4):639–45. doi:10.1080/17470210903469864 PubMed PMID: 20017058.

10.	van der Wel P, van Steenbergen H. Pupil dilation as an index of effort in cognitive control tasks: A review. Psychon Bull Rev. 2018 Dec 1;25(6):2005–15. doi:10.3758/s13423-018-1432-y

11.	Kelbsch C, Strasser T, Chen Y, Feigl B, Gamlin PD, Kardon R, et al. Standards in Pupillography. Front Neurol. 2019 Feb 22;10. doi:10.3389/fneur.2019.00129
